# Supplementary material for: Glycogen branching enzyme controls cellular iron homeostasis via Iron Regulatory Protein 1 and mitoNEET
Source: Nat Commun. 2019 Nov 29;10:5463. doi: 10.1038/s41467-019-13237-8 (PMC6884552; doi:10.1038/s41467-019-13237-8)
Supplement: Supplementary file 1 — Supplementary Information [file 41467_2019_13237_MOESM1_ESM.pdf]

Supplementary Information

“Glycogen Branching Enzyme controls cellular iron homeostasis via Iron Regulatory Protein 1 and mitoNEET”

Huynh et al.

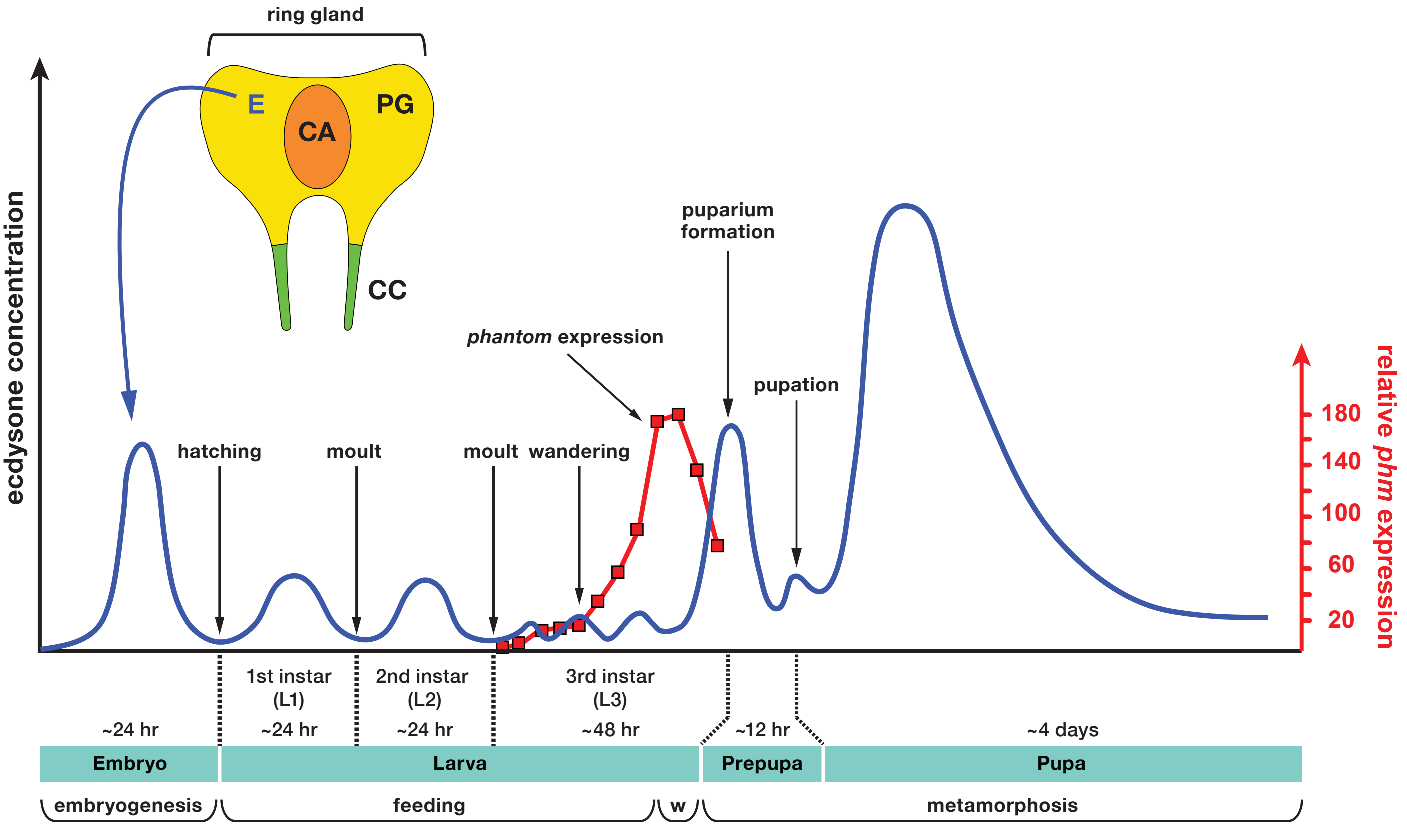

Supplementary Figure 1. Ecdysone production during *Drosophila* development. Ecdysone (E) is produced in the prothoracic gland (PG). The ring gland is an endocrine tissue composed of three fused glands: The PG, the CA (corpus allatum) and the CC (corpora cardiaca). Pulses of ecdysone trigger major developmental transitions such as hatching, the moults, wandering behaviour, and the onset of metamorphosis. The relative expression profile of one of the Halloween genes with PG-specific expression, *phantom*, is shown in red, and is based on qPCR results for 12 time points ranging from 4 hr to 48 hr after the L2/L3 moult, covering the entirety of the L3 stage (48 hr). While absolute phantom (*phm*) transcript levels are already very high at 4 hr after the L2/L3 moult (4 hr time point normalized to 1), expression of the gene continues to rise until the 40 hr time point in preparation of the late L3 pulse that triggers puparium formation. Phantom is a cytochrome P450 enzyme and requires haem as a cofactor, indicating that iron and haem requirements of the PG change dynamically, depending on the developmental time point.

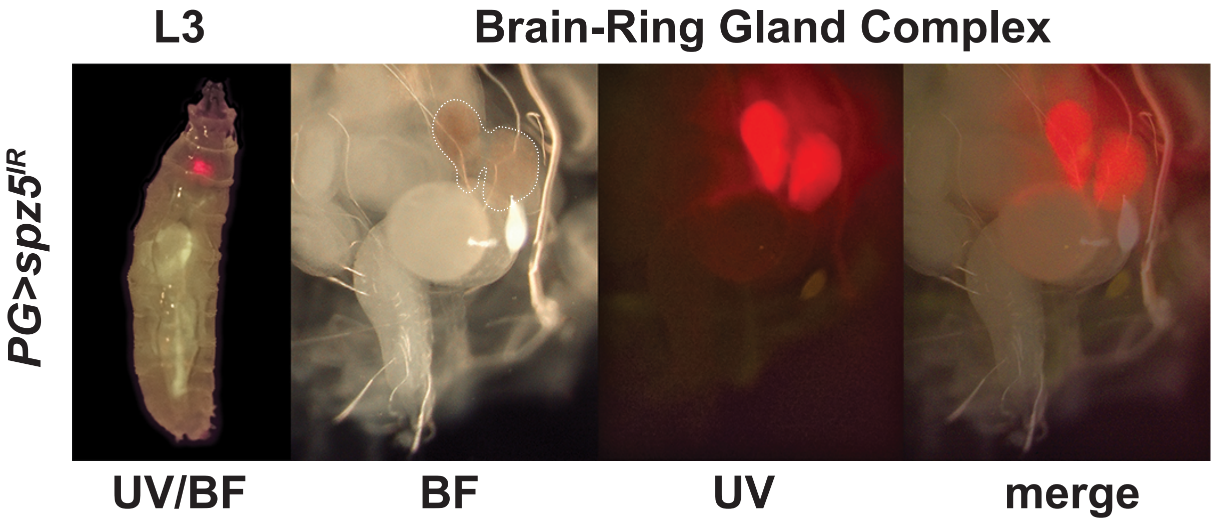

Supplementary Figure 2. Porphyria-like phenotype in the *Drosophila* prothoracic gland. Red autofluorescence is visible in whole larvae when exposed to ultraviolet (UV) light (left). Dissection shows an enlarged ring gland of red-brownish colour (dotted line) in brightfield (BF) light. The three glands that comprise the ring gland are not discernible in this image, but RNAi expression is limited to the prothoracic gland (PG). "*PG>*" refers to the "*phm22-Gal4*" driver, which mediates PG-specific expression. Pictures show results for VDRC line #102389 targeting the *spatzle5* gene (*spz5*^{IR}). Wild type controls not shown. UV exposure shows autofluorescence caused by accumulated protoporphyrins.

Haem biosynthesis	gene name (human)	Associated disorder	gene name (Drosophila)
Succinyl-CoA + Glycine → ALA	*ALAS*	X-linked sideroblastic anemia	*Alas*
ALA → Porphobilinogen	*ALAD*	ALAD porphyria (Doss porphyria)	*Pbgs*
Porphobilinogen → Hydroxymethylbilane	*PBGD*	Acute intermittent porphyria	*l(3)02640*
Hydroxymethylbilane → Uroporphyrinogen III	*UROS*	Congenital erythroipoietic porphyria (Gunther disease)	*CG1885*
Uroporphyrinogen III → Coproporphyrinogen III	*UROD*	Porphyria cutanea tarda	*Updo*
Coproporphyrinogen III → Protoporphyrinogen IX	*CPOX*	Coproporphyria	*Coprox*
Protoporphyrinogen IX → Protoporphyrin IX	*PPOX*	Variegate porphyria	*Ppox*
Protoporphyrin IX + iron → Haem	*FECH*	Erythropoietic porphyria	*Fech*

Supplementary Figure 3. Haem pathway and associated human disorders. Haem is produced from glycine and Succinyl-CoA through a series of eight enzymatic steps, and highly conserved in all animals. Enzyme names are abbreviated: ALA Synthase (ALAS); ALA Dehydratase (ALAD); Porphobilinogen Deaminase (PBGD); Uroporphyrinogen III Synthase (UROS); Uroporphyrinogen III Decarboxylase (UROD); Coproporphyrinogen III oxidase (CPOX); Protoporphyrinogen IX oxidase; and Ferrochelatase (FECH).

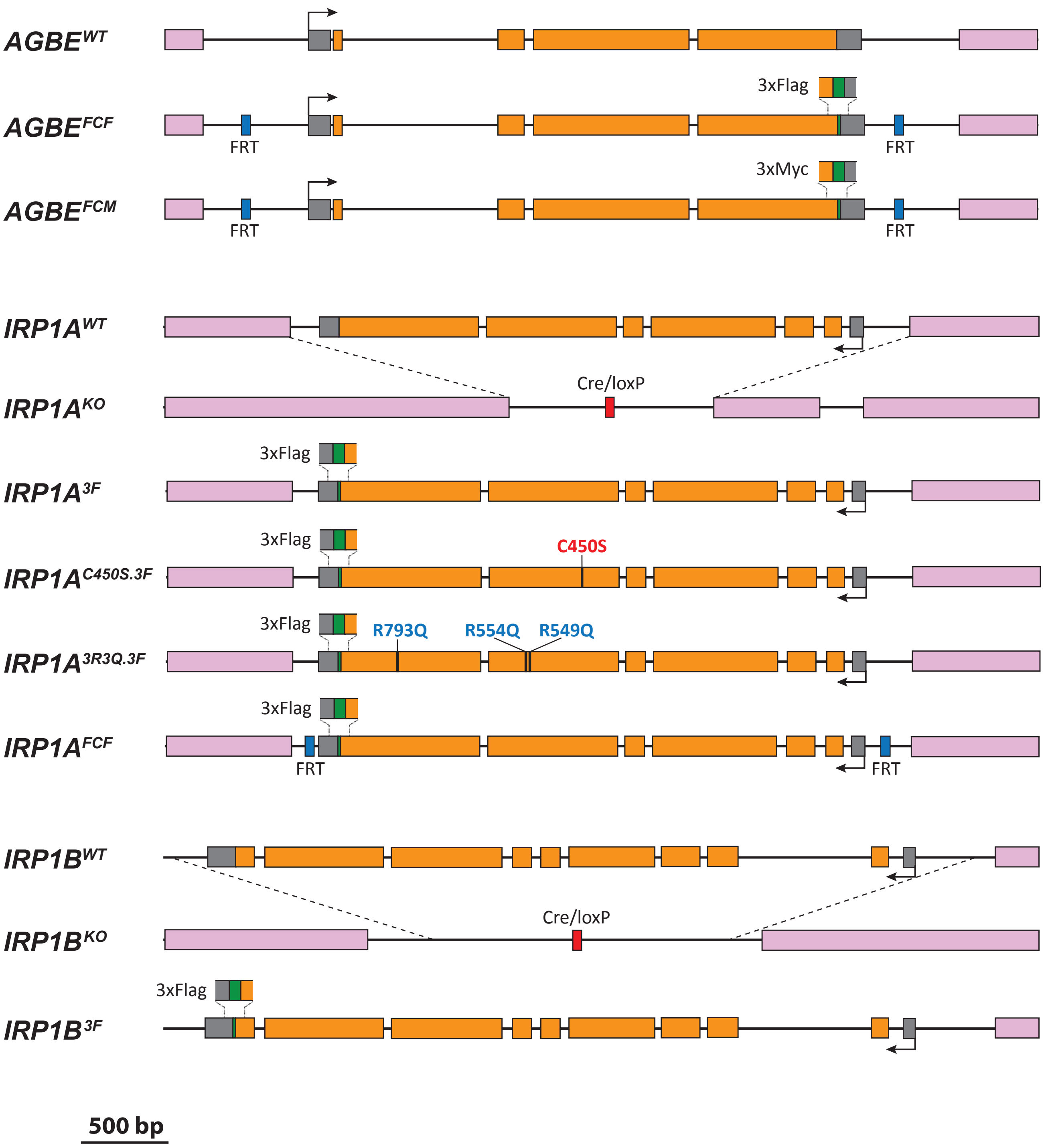

Neighbouring gene(s)

Flippase (FLP) Recombinase Target

Untranslated Region

Translated Exon of target gene

Epitope tag

Cre/loxP site

C450S: point mutation (C ⟶ S) causing 1) constitutive RNA-binding of IRP1A and 2) loss of IRP1A aconitase function.

RXXXQ: Three point mutations (R ⟶ Q), which together cause strongly reduced RNA-binding of IRP1A. Aconitase activity intact.

Huynh et al., Supplementary Figure 4

◄ **Supplementary Figure 4. CRISPR/CAS9-mediated knock-in lines.** We targeted three genes, *AGBE*, *IRP1A*, and *IRP1B*, each of which encodes a single mRNA isoform, respectively. Allele naming: WT: wild type; FCF: FRT/CRISPR/3xFlag; FCM: FRT/CRISPR/3xMyc. KO: knockout. 3F: 3xFlag. Note: Homozygous $IRP1A^{FCF}$ flies die on normal fly medium, but are viable when the diet is supplemented with iron, indicating that the FRT site insertions disrupt IRP1A function. Excision of *IRP1A* via prothoracic gland-specific expression of Flippase (FLP) caused protoporphyrin accumulation when second instar larvae were transferred from iron-supplemented to iron-depleted food (see Fig. 3B).

pathway & metabolites	vertebrate enzyme	fly enzyme	PG phenotype	WB phenotype
Glucose				
↓	Hexokinase	Hex A (CG3001)	10-20% pupal lethal	embryonic lethal
Glucose-6-phosphate				
↓	Phospho glucomutase	Pgm (CG5165)	normal	embryonic lethal
Glucose-1-phosphate				
↓	UDP-glucose pyrophosphorylase	UGP (CG4347)	normal	L2 lethal
Uracil-diphosphate glucose				
↓	Glycogen synthase	GlyS (CG6904)	normal	L2 & L3 lethal
↓	Glycogen Branching Enzyme (GBE1)	AGBE (CG33138)	96% L3 lethal fluorescent PG	embryonic & L1 lethal
Glycogen				

Supplementary Figure 5. Phenotypes associated with prothoracic gland-specific and ubiquitous depletion of enzymes acting in the glycogen biosynthetic pathway of *Drosophila*. Names for vertebrate and *Drosophila* enzyme orthologues are shown in blue. PG = prothoracic gland-specific expression (*phm22-Gal4* x *UAS*-RNAi). WB = whole body expression (*tubulin-Gal4* x *UAS*-RNAi). Red indicates the appearance of red autofluorescence in the *Drosophila* prothoracic gland.

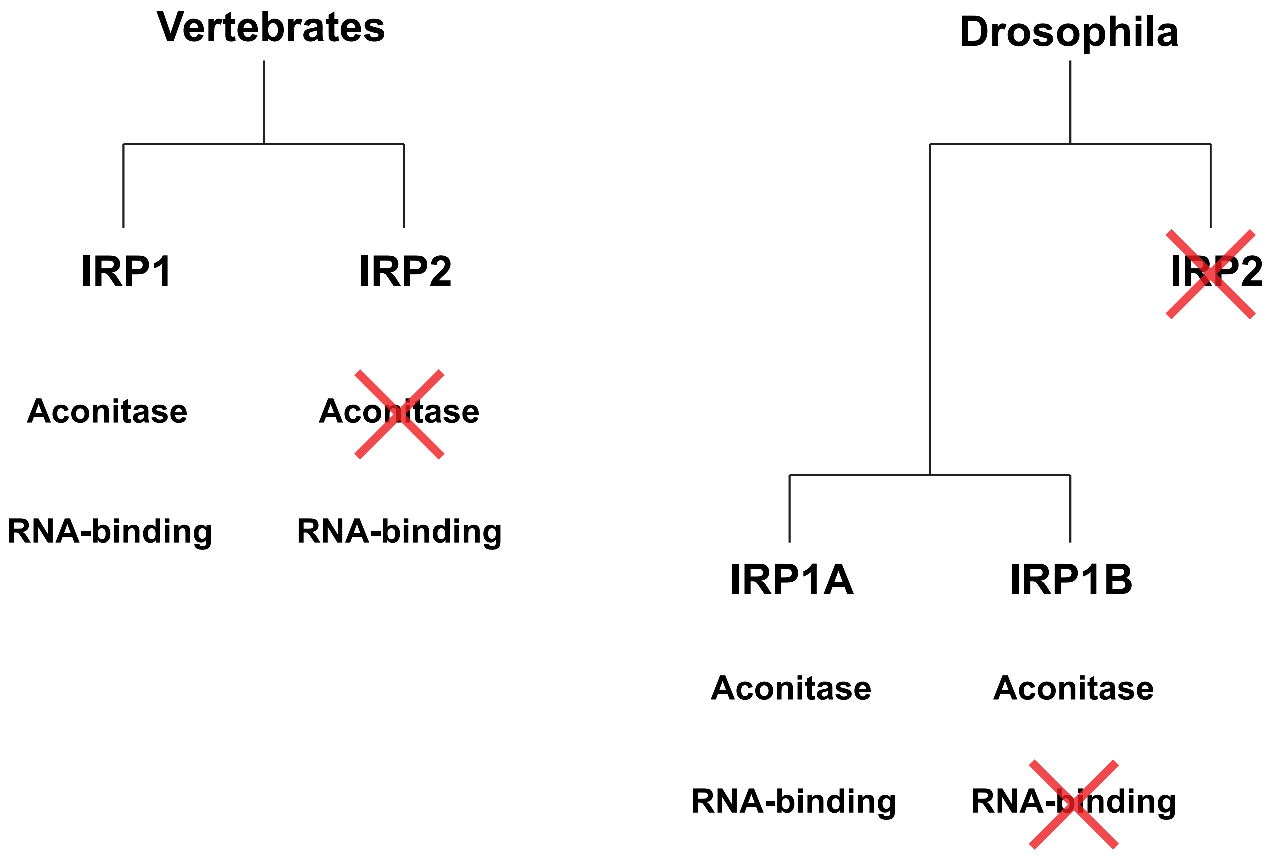

Supplementary Figure 6. Comparison of genes encoding Iron-Regulatory Proteins (IRPs) between vertebrates and *Drosophila melanogaster.* Flies lack IRP2, but have two IRP1 genes. Only IRP1A was shown to bind to canonical Iron Responsive Elements in target mRNAs.

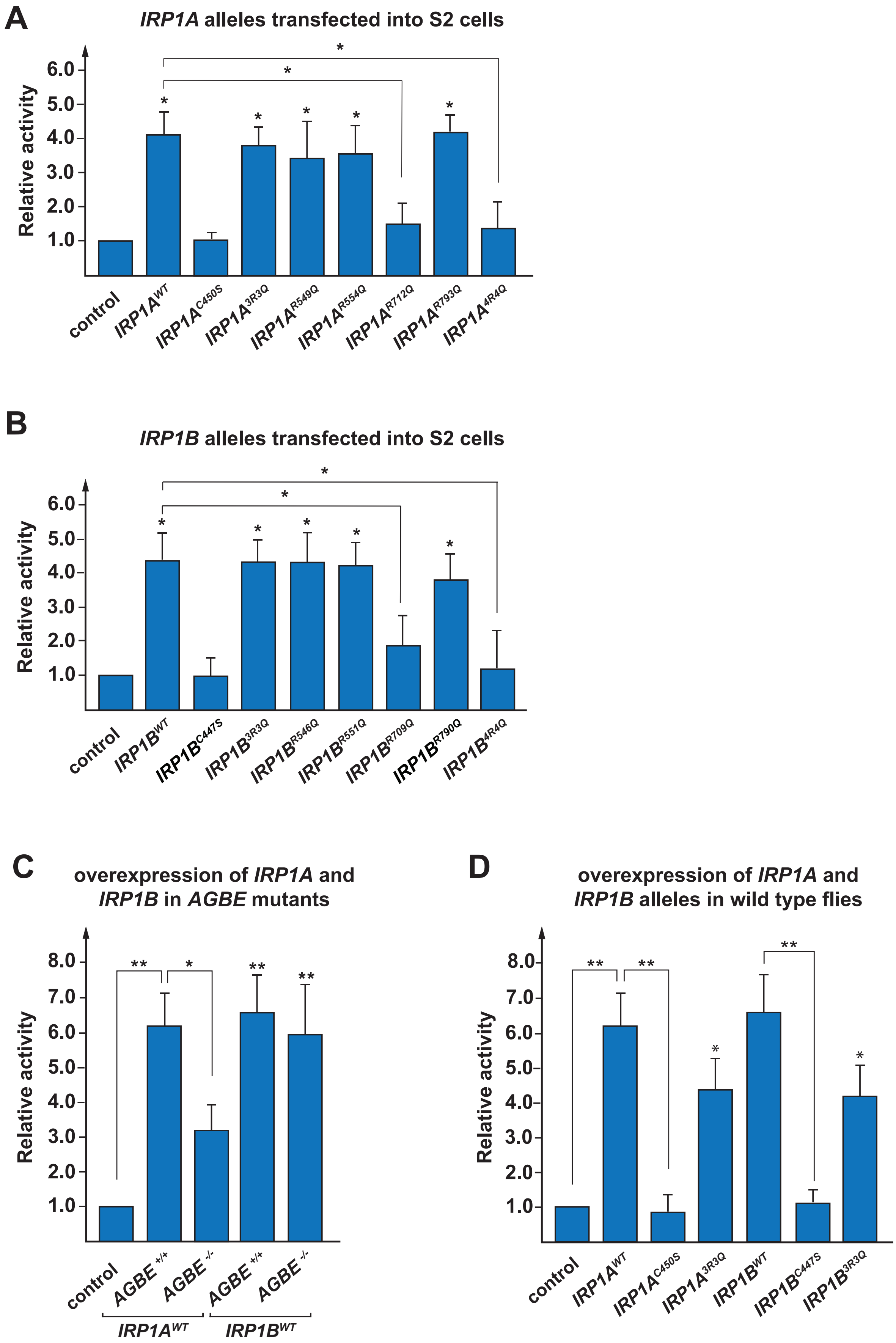

Huynh et al., Supplementary Figure 7

◄ **Supplementary Figure 7. Aconitase activity of IRP1 variants. A-B.** Relative aconitase activity of IRP1A (A) and IRP1B (B) variants in S2 cell culture. Results were normalized to untransfected S2 cells. Transfection efficiency was evaluated by SDS-PAGE to normalize protein levels for aconitase assay (data not shown). IRP1A^{3R3Q}: non-RNA binding (Fig. 2C), has amino acid substitutions (R→Q) in positions 549, 554 and 793. IRP1A^{4R4Q} : predicted to be non-RNA binding, has amino acid substitutions (R→Q) in positions 549, 554, 712 and 793. IRP1A^{C450S}: constitutively RNA-binding, has amino acid substitution (C→S) in position 450. The IRP1B^{3R3Q}, IRP1B^{4R4Q} and IRP1B^{C447S} variants have corresponding substitutions in IRP1B**. C.** Relative aconitase activity of whole body extracts from first instar larvae (L1) that ubiquitously expressed *IRP1A* or *IRP1B* in either wild type or *AGBE*^{FCF} (Supplementary Fig. 4) mutant backgrounds. Ubiquitous excision of *AGBE* results in L1 lethality. Data were normalized to control animals that lack *IRP1* transgenes. **D.** Relative aconitase activity of whole body extracts from first instar larvae (L1) that ubiquitously expressed one of the following transgenes in a wild type background: *IRP1A*^{WT}, *IRP1A*^{C450S}, *IRP1A*^{3R3Q}, *IRP1B*^{WT}, *IRP1B*^{C447S} and *IRP1B*^{3R3Q}. For allele properties, see A-B. **A-D.** Asterisks indicate a *P*-value <0.05 (*) or <0.01 (**) relative to the control, or relative to the indicated reference sample. Source data are provided as a Source Data file.

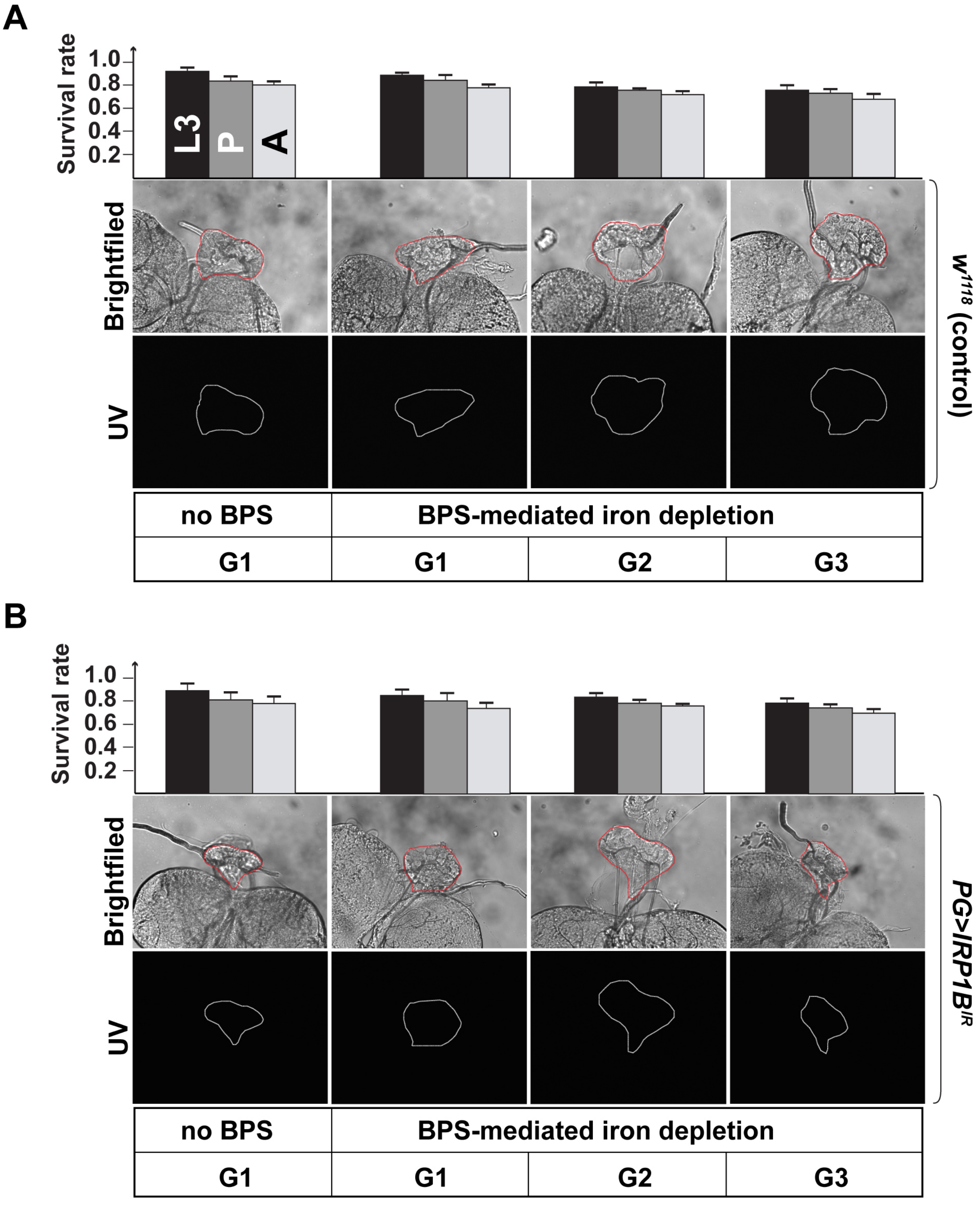

Supplementary Fig. 8. Ring gland phenotypes in control and *IRP1B*^{IR} lines are normal when raised for three generations on iron-depleted media. Control flies (w^{1118}) (A) and PG-specific *IRP1B*-RNAi (*PG>IRP1B*^{IR}) animals (B) were kept continuously on Bathophenanthroline Sulfate (BPS)-containing food to deplete cellular iron stores over three consecutive generations. Third instar (L3, black), pupal (P, dark gray) and adult (A, light gray) survival was scored for each generation. Ring glands were dissected during the L3 stage and examined under brightfield and UV light. Source data are provided as a Source Data file.

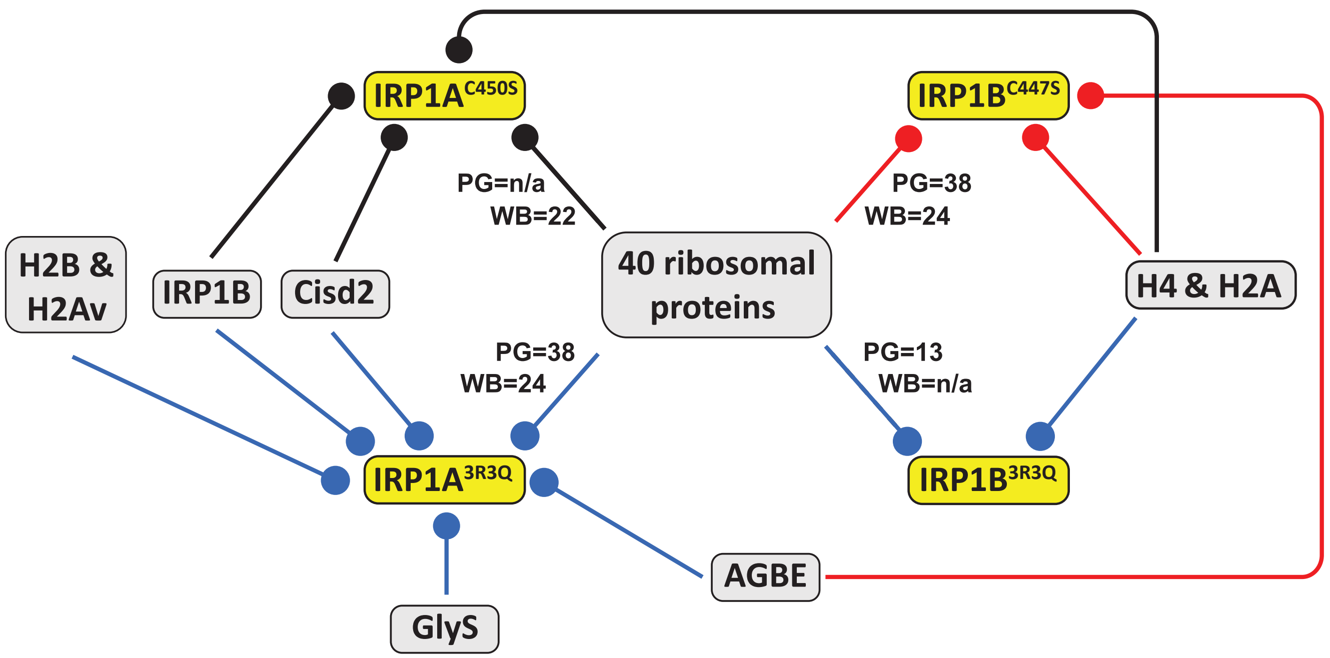

Supplementary Figure 9. Interaction map for transgenic *IRP1A* and *IRP1B* lines. C450S: abolishes Fe-S cluster incorporation into IRP1A. 3R3Q: Replacement of three arginines implicated in RNA-binding (Supplementary Table 1). Red: Interaction detected in prothoracic gland (PG) and whole body (WB) samples. Black: Detected in WB samples only. Blue: Tested in PG samples only. Yellow boxes represent transgenically expressed 3xFlag-tagged protein that was immunoprecipitated with anti-Flag antibodies. Circles next to yellow boxes indicate whether the interaction was reciprocal (circles on both ends) or not (circle on one end). In total, 40 ribosomal proteins were common to all tested PG and WB sets (see Supplementary Tables 3-4), numbers indicate how many were detected in either PG or WB samples. H4, H2A, H2av and H2B are histone proteins. Cisd2 = mitoNEET, GlyS = Glycogen Synthase, AGBE = 1,4-Alpha-Glucan Branching Enzyme.

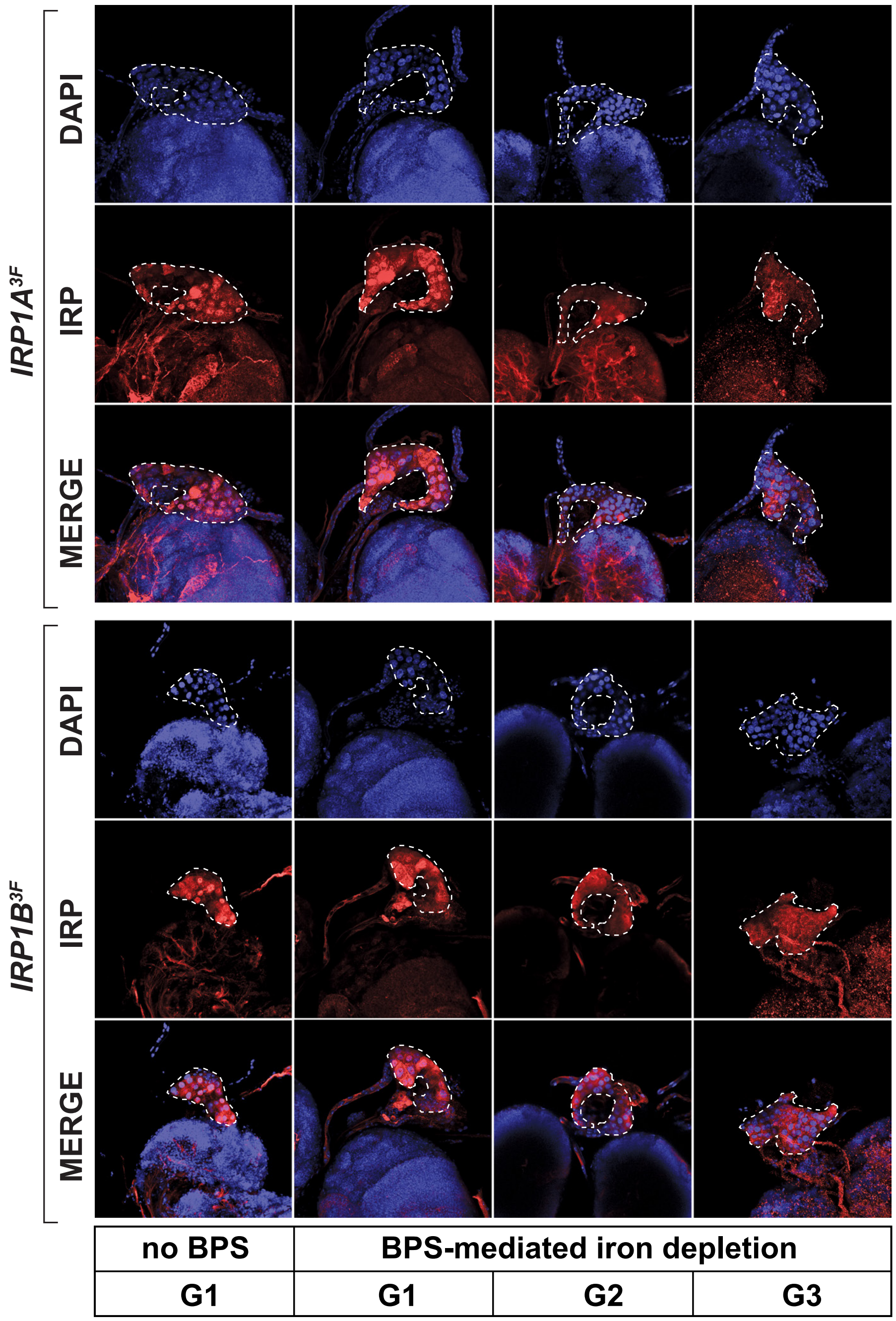

Supplementary Figure 10. Nuclear localization of IRP1A and IRP1B in the prothoracic gland when reared under sustained iron-depleted conditions. Flies were reared on Bathophenanthroline Sulfate (BPS)-supplemented media for three generations. Each generation (G1-G3), ring glands were stained for the subcellular localization of Flag-tagged proteins produced from knock-in alleles of *IRP1A* and *IRP1B* (*IRP1A*^{3F} and *IRP1B*^{3F}, Supplementary Fig. 4). DAPI was used to stain DNA/nuclei.

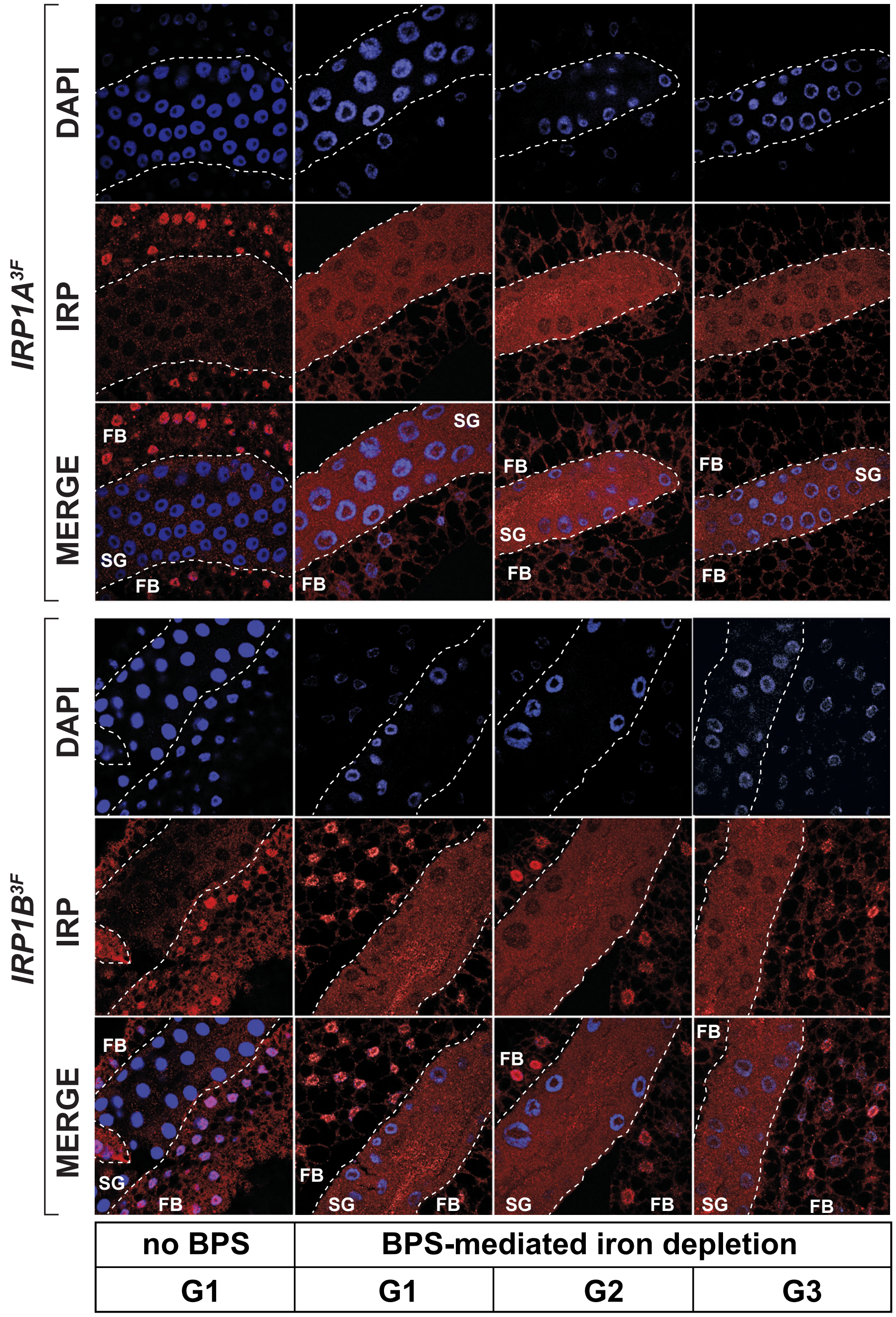

Supplementary Figure 11. Nuclear localization of IRP1A and IRP1B in salivary gland and fat body tissue when reared under sustained iron-depleted conditions. Flies were reared on Bathophenanthroline Sulfate (BPS)-supplemented media for three generations. Each generation (G1-G3), ring glands were stained for the subcellular localization of Flag-tagged proteins produced from knock-in alleles of *IRP1A* and *IRP1B* (*IRP1A*^{3F} and *IRP1B*^{3F}, Supplementary Fig. 4). DAPI was used to stain DNA/nuclei. SG: salivary gland. FB: fat body.

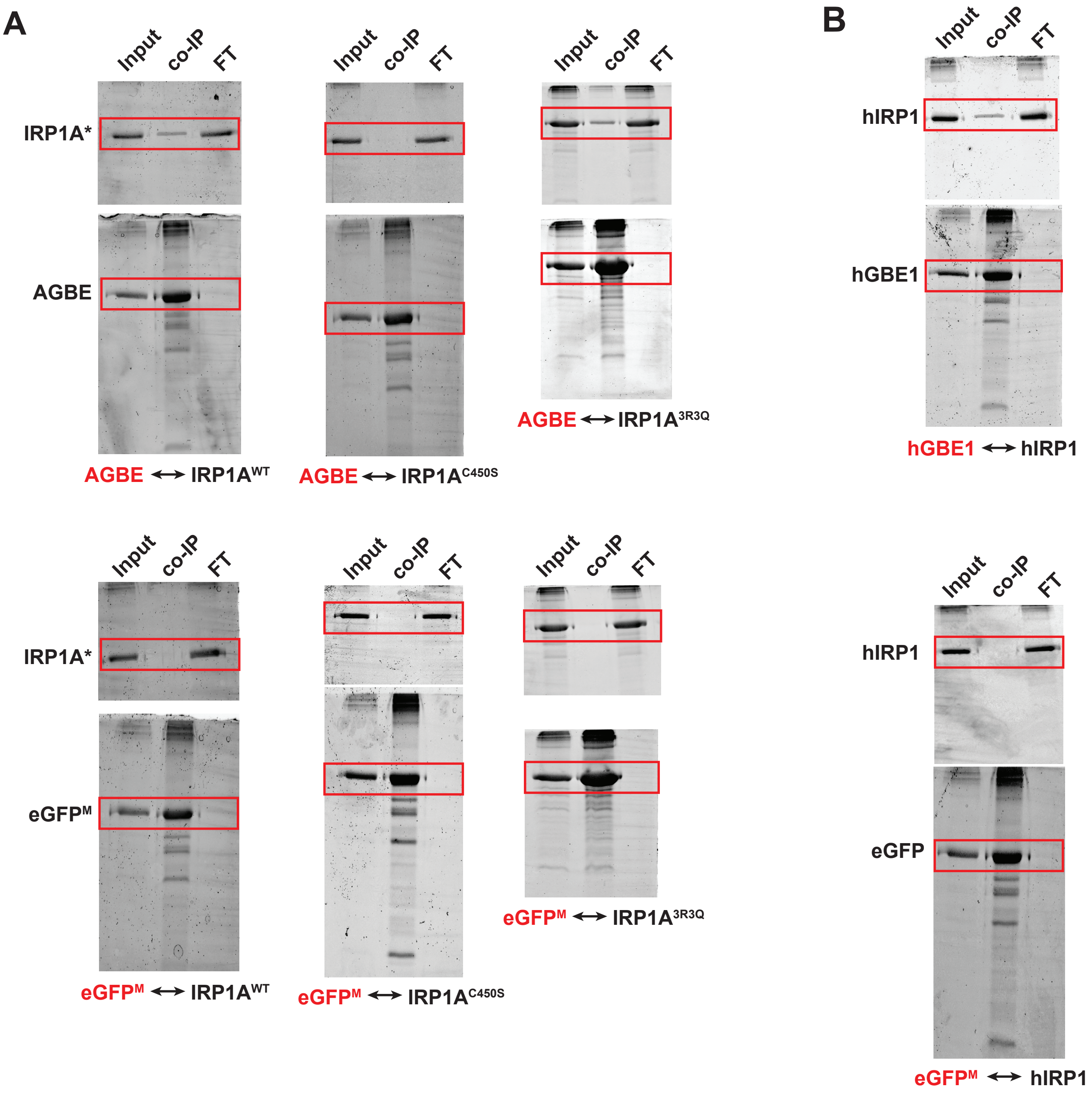

Supplementary Figure 12. Uncropped Western Blots corresponding to Fig. 2. A. Uncropped Western Blots corresponding to Fig. 2A. **B.** Uncropped Western Blots corresponding to Fig. 2B.

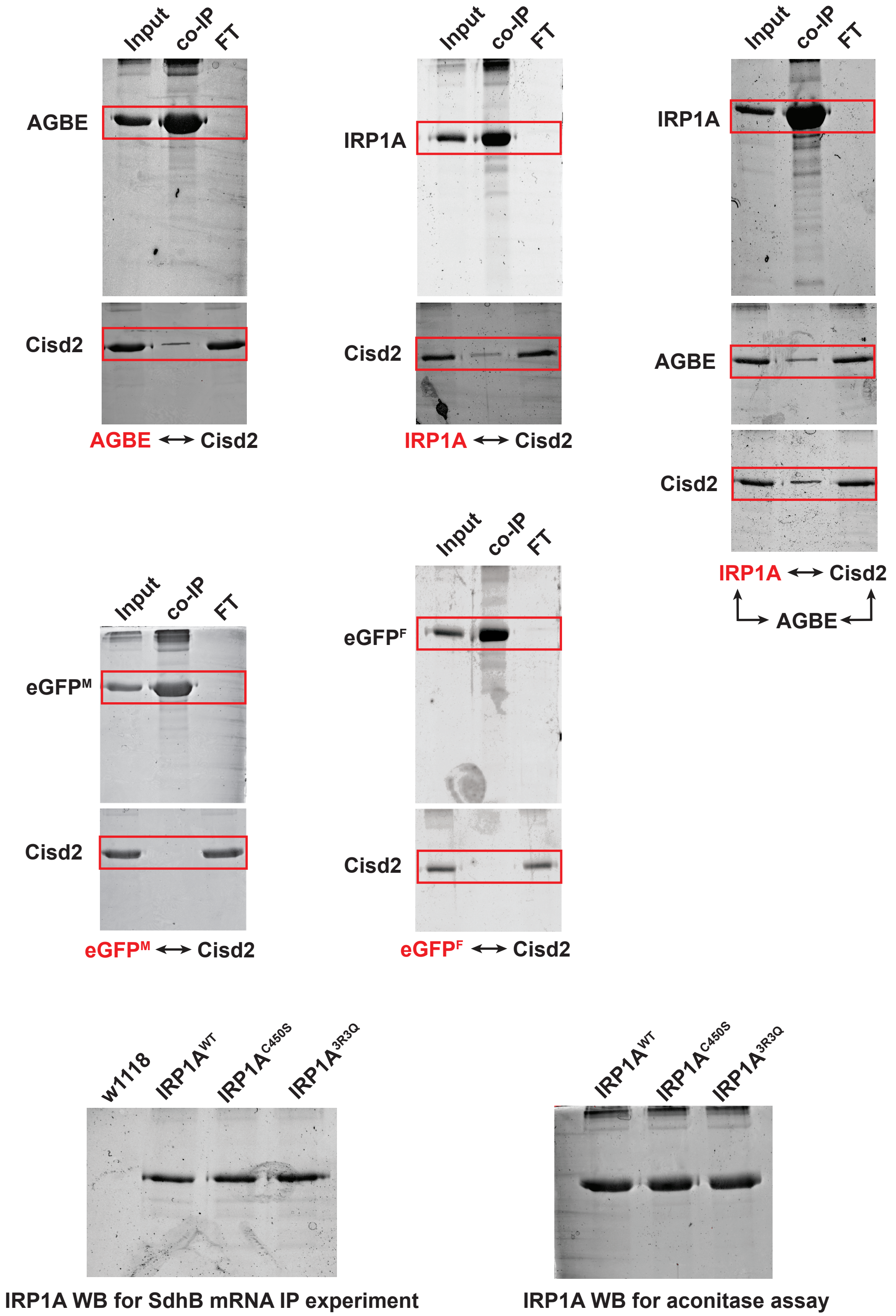

Supplementary Figure 13. Uncropped Western Blots corresponding to Fig. 4.

Supplementary Table 1. Transgenic constructs and properties.

**transgene**	**Description**
UAS-3xFlag-IRP1A^{WT}	Expresses wild type 3xFlag-tagged *IRP1A* cDNA under *Gal4/UAS* control.
UAS-IRP1A^{WT}	Expresses wild type untagged *IRP1A* cDNA under Gal4/UAS control.
UAS-3xFlag-IRP1A^{C450S}	Expresses mutant 3xFlag-tagged *IRP1A*^{C450S} cDNA under *Gal4/UAS* control, single point mutation abolishes Fe-S-binding and forces protein to assume apo-form[1,2]. Predicted to abolish aconitase function and render protein constitutively RNA-binding.
UAS-IRP1A^{C450S}	Expresses mutant untagged *IRP1A*^{C450S} cDNA under *Gal4/UAS* control, single point mutation abolishes Fe-S-binding and forces protein to assume apo-form[1,2]. Predicted to abolish aconitase function and render protein constitutively RNA-binding.
UAS-3xFlag-IRP1A^{3R3Q}	Expresses mutant 3xFlag-tagged *IRP1A*^{3R3Q} cDNA under *Gal4/UAS* control, converting three arginine into three glutamine residues (R549Q, R554Q and R793Q). Predicted to result in non-RNA-binding holo-IRP1A[1,2].
UAS-3xFlag-IRP1B^{WT}	Expresses wild type 3xFlag-tagged *IRP1B* cDNA under *Gal4/UAS* control.
dU6-3-IRP1A^{gRNA}	ubiquitously expresses two *IRP1A* gRNAs for somatic disruption when crossed to CAS9-expressing stock[3] (PG-specific CAS9, causes red autofluorescence).
UAS-IRP1B^{WT}	Expresses wild type untagged *IRP1B* cDNA under *Gal4/UAS* control.
UAS-3xFlag-IRP1B^{C447S}	Expresses mutant 3xFlag-tagged *IRP1B*^{C447S} cDNA under *Gal4/UAS* control, mutation at the same cysteine residue as in *IRP1A*^{C450S}.
UAS-IRP1B^{C447S}	Expresses mutant untagged *IRP1B*^{C447S} cDNA under *Gal4/UAS* control, single point mutation affecting equivalent cysteine residue as in *IRP1A*^{C450S}. Predicted to abolish aconitase function.
UAS-3xFlag-IRP1B^{3R3Q}	Expresses mutant 3xFlag-tagged IRP1B^{3R3Q} cDNA under *Gal4/UAS* control, point mutations equivalent to those in IRP1A^{3R3Q} . Predicted to interfere with RNA-binding, however, no RNA-binding has been reported for IRP1B.
UAS-Yeast Aco1^{WT}	Expresses wild type untagged yeast *Aco1* cDNA under *Gal4/UAS* control, predicted to cause cytoplasmic and mitochondrial localization of the enzyme[3].
UAS-Yeast Aco1$^{\Delta Sp}$	Expresses mutant untagged yeast *Aco1* cDNA under *Gal4/UAS* control. The mutation removes the mitochondrial targeting sequence[4].
UAS-3xFlag-hIRP1^{WT}	Expresses 3xFlag-tagged human *IRP1* cDNA under *Gal4/UAS* control.
UAS-3xFlag-hIRP2^{WT}	Expresses 3xFlag-tagged human *IRP2* cDNA under *Gal4/UAS* control.

Supplementary Table 2. Samples tested in Mass spectrometry.					
**genotype**	**allele type**	**prothoracic gland (regular fly food)**	**whole body (regular fly food)**	**whole body (BPS - G1)**	**whole body (BPS - G2)**
w^{1118}	wild type	2 samples	1 sample	1 sample	1 sample
IRP1A^{3F}	knock-in	1 sample	2 samples	1 sample	1 sample
IRP1A^{C450S}	transgene	not tested	2 samples	not tested	not tested
IRP1A^{3R3Q}	transgene	1 sample	not tested	not tested	not tested
IRP1B^{3F}	knock-in	1 sample	1 sample	not tested	not tested
IRP1B^{C447S}	transgene	1 sample	1 sample	not tested	not tested
IRP1B^{3R3Q}	transgene	1 sample	1 sample	not tested	not tested
AGBE^{FCF}	knock-in	not tested	2 samples	not tested	not tested

Table S2. Samples tested in mass spectrometry (MS). All *AGBE* and *IRP1A/B* alleles, with the exception of those in the control line *w^{1118}*, were Flag-tagged. The transgenes were under *UAS*-control, allowing expression with a prothoracic gland-specific Gal4 driver (*phm22-Gal4* = *PG-Gal4*). Transgenic whole-body samples were based on the *tubulin-Gal4* driver, which mediates ubiquitous expression. Prothoracic glands (PG) also expressed a CD8 membrane marker for affinity-purification of isolated PG cells from 10,000-20,000 hand-dissected Brain-Ring Gland complexes (see Materials and Methods). Knock-in alleles were controlled by their endogenous regulatory regions. ***IRP1A^{3F}***, ***IRP1B^{3F}*:** last exon of endogenous alleles have been replaced with flag-tagged exon (Supplementary Figure 4).
AGBE^{FCF}: endogenous alleles have been replaced with FRT-flanked version, last exon flag-tagged (Supplementary Figure 4). ***IRP1A^{C450S}*** and ***IRP1B^{C447S}***: Flag-tagged, and carry a single point mutation that abolishes Fe-S binding, rendering IRP1A constitutively RNA-binding, and abolishing IRP1B aconitase activity (Supplementary Table 1). *IRP1A^{3R3Q}*, *IRP1B^{3R3Q}*: Replacement of three arginines implicated in RNA-binding with three glutamines, abolishes RNA-binding of IRP1A (Figure 2C). For four conditions, we tested two samples (biological replicates), and reproducibility of results ranged from 82.4-98.4% (data not shown). See Supplementary Table 3 for all results.

Supplementary References (relate to Supplemental Table 1):

1. Philpott, C. C., Klausner, R. D., & Rouault, T. A. (1994). The bifunctional iron-responsive element binding protein/cytosolic aconitase: The role of active-site residues in ligand binding and regulation. *Proceedings of the National Academy of Sciences, 91*(15), 7321-7325.

2. Walden, W. E., Selezneva, A. I., Dupuy, J., Volbeda, A., Fontecilla-Camps, J. C., Theil, E. C., & Volz, K. (2006). Structure of Dual Function Iron Regulatory Protein 1 Complexed with Ferritin IRE-RNA. *Science, 314*(5807), 1903-1908.

3. Huynh, N., Zeng, J., Liu, W., & King-Jones, K. (2018). A Drosophila CRISPR/Cas9 Toolkit for Conditionally Manipulating Gene Expression in the Prothoracic Gland as a Test Case for Polytene Tissues. G3 (Bethesda, Md.), 8(11), 3593–3605. http://doi.org/10.1534/g3.118.200539

4. Regev-Rudzki, N. (2005). Yeast Aconitase in Two Locations and Two Metabolic Pathways: Seeing Small Amounts Is Believing. *Molecular Biology of the Cell, 16*(9), 4163-4171.
