# Supplementary material for: Glycogen branching enzyme controls cellular iron homeostasis via Iron Regulatory Protein 1 and mitoNEET
Source: Nat Commun. 2019 Nov 29;10:5463. doi: 10.1038/s41467-019-13237-8 (PMC6884552; doi:10.1038/s41467-019-13237-8)
Supplement: Supplementary file 3 — Description of Additional Supplementary Files [file 41467_2019_13237_MOESM3_ESM.pdf]

Description of Additional Supplementary Files

File Name: Supplementary Data 1
Description: Mass spectrometry results for ring gland and whole body samples.

File Name: Supplementary Data 2
Description: Gene ontology analysis of mass spectrometry results.

File Name: Supplementary Data 3
Description: RNA-Seq data listing 234 genes downregulated by transgenic expression of IRP1A variants.

File Name: Supplementary Data 4
Description: Gene ontology analysis of 234 downregulated genes obtained from RNASeq results.
